# Poly(POG)n loaded with recombinant human bone morphogenetic protein-2 accelerates new bone formation in a critical-sized bone defect mouse model

**DOI:** 10.1186/s13018-020-01977-z

**Published:** 2020-10-14

**Authors:** Ryo Tazawa, Kentaro Uchida, Hiroaki Minehara, Terumasa Matsuura, Tadashi Kawamura, Hiroyuki Sekiguchi, Kyoko Muneshige, Sho Inoue, Gen Inoue, Masashi Takaso

**Affiliations:** 1 Department of Orthopaedic Surgery, Kitasato University School of Medicine, 1-15-1 Kitasato, Minami-ku, Sagamihara City, Kanagawa 252-0374 Japan; 2 Shonan University of Medical Sciences Research Institute, Nishikubo 500, Chigasaki City, Kanagawa 253-0083 Japan

**Keywords:** Collagen-like peptides, Poly(POG)n, Bone defect, New bone formation, Bone morphogenetic protein-2

## Abstract

**Background:**

Delivery of bone morphogenetic protein-2 (BMP-2) via animal-derived absorbable collagen materials is used for the treatment of large bone defects. However, the administration of bovine proteins to humans is associated with the risk of zoonotic complications. We therefore examined the effect of combining BMP-2 with collagen-like peptides, poly(POG)n, in a critical-sized bone defect mouse model.

**Methods:**

A 2-mm critical-sized bone defect was created in the femur of 9-week-old male C57/BL6J mice. Mice were randomly allocated into one of four treatment groups (*n* = 6 each): control (no treatment), poly(POG)n only, 0.2 μg, or 2.0 μg BMP-2 with poly(POG)n. New bone formation was monitored using soft X-ray radiographs, and bone formation at the bone defect site was examined using micro-computed tomography and histological examination at 4 weeks after surgery.

**Results:**

Administration of 2.0 μg of BMP-2 with poly(POG)n promoted new bone formation and resulted in greater bone volume and bone mineral content than that observed in the control group and successfully achieved consolidation. In contrast, bone formation in all other groups was scarce.

**Conclusions:**

Our findings suggest the potential of BMP-2 with poly(POG)n as a material, free from animal-derived collagen, for the treatment of large bone defects.

## Background

There are significant clinical limitations to the rebuilding of large bone segments following extensive bone loss as result of pathological events including trauma, inflammation, and surgical treatment of tumors. Recombinant human (rh) bone morphogenetic protein (BMP)-2 has immense potential in clinical applications. However, it has a short half-life, is expensive, and is associated with the risk of heterotopic ossification [1–6]. Therefore, the development of a carrier is needed for the retention of rhBMP-2 to enhance bone formation and reduce side effects in clinical settings.

Absorbable collagen sponge (ACS), derived from bovine collagen type I, has been developed to increase the retention of rhBMP-2, and a ACS/rhBMP-2 composite has been shown to accelerate bone healing in bone defects in several animal models [7–10]. ACS/rhBMP-2 is currently used for the treatment of open fractures and spinal surgery in the USA [11–13]. However, the use of animal-derived collagen is associated with potential zoonotic complications, particularly the transmission of unknown infectious diseases such as bovine spongiform encephalitis [14].

The amino acid sequence POG typically makes up fibrous collagen, and poly(POG)n has been extensively used as a model collagen peptide due to its stable triple helix structure [15]. Given its collagen-like structure, thermal stability, and pathogen-free nature, poly(POG)n has been examined as a biomaterial for skin regeneration [16]. In addition, there is no risk of zoonotic complications with non-animal-derived collagen-like peptides. These characteristics indicate that poly(POG)n may be a suitable alternative to ACS as a carrier for BMP-2, although the efficacy of poly(POG)n gels as a carrier for rhBMP-2 in the treatment of bone defects remains to be determined.

Here, we investigated the efficacy of rhBMP-2 combined with a gel formed by poly(POG)n, which is free from animal-derived collagen, in a critical-sized bone defect mouse model.

## Methods

### Chemicals

The collagen-like polypeptide poly(POG)n gel was purchased from JNC Corporation (Tokyo, Japan), and rhBMP-2 from PeproTech Inc. (Rocky Hill, NJ, USA). Homology of the amino acid sequence of native BMP-2 is 91% between humans and mice. However, homology of the amino acid sequence of rhBMP-2 protein (QAKHKQRKRLKSSCKRHPLYVDFSDVGWNDWIVAPPGYHAFYCHGECPFPLADHLNSTNHAIVQTLVNSVNSKIPKACCVPTELSAISMLYLDENEKVVLKNYQDMVVEGCGCR) is 100%. Therefore, we used rhBMP-2 in our study. To prepare graft materials, 2.5 μl of 2 μg/μl rhBMP-2 solution or PBS solution was added to 22.5 μl of 1% poly(POG)n gel and vortexed gently before administration.

### External fixation device

MouseExFix Simple L® (Research Implant System, RIS, Davos, Switzerland), an external fixation device consisting of a fixator block and four 0.45-mm-diameter mounting pins, was used to stabilize femur bones after creating the defect.

### Animals

All surgeries and handling procedures were conducted in accordance with the guidelines of the Animal Ethics Committee of Kitasato University (permission number 2018-087). A total of 24 9-week-old male C57BL/6 J mice (Charles River Laboratories Japan, Inc., Yokohama, Japan) were used for this experiment. The mice were given standard laboratory chow (CRF-1, Oriental Yeast, Tokyo, Japan) and kept in a room with controlled temperature (23 ± 2 °C) and humidity (55 ± 10%) and a 12-h light/dark cycle.

All mice received a 2-mm bone defect created in the center of the right femur and fixed with MouseExFix Simple L® (see details below) before receiving treatment according to their group allocation. Mice were randomly allocated to one of four treatment groups, with 6 mice in each group: group CONT (control group) did not receive any chemical treatment for the bone defect; group GEL received 25 μl of poly(POG)n only; group BMP(0.2) received 25 μl of poly(POG)n with 0.2 μg rhBMP-2; and group BMP(2.0) received 25 μl of poly(POG)n with 2.0 μg rhBMP-2.

### Surgical procedure

All mice were initially sedated using isoflurane followed by an intramuscular injection of a Vetorphale, Domitor, and Midazolam mixture at 1:3:1 (0.075 ml/100 g). The bone defect was created in only the right femur. The surgical site was prepared preoperatively by removing the surrounding fur and sterilizing the underlying skin. Under aseptic conditions, a 20-mm longitudinal incision was made along the lateral femur from the hip to the knee. The fascia latae was cut open between the gluteus superficialis and biceps femoris muscles to expose the femur. The femur was fixed using MouseExFix Simple L® such that the device was centrally positioned, parallel to the longitudinal axis of the femur. After predrilling, the first mounting pin was inserted through the most distal hole in the fixator block, and the second mounting pin was inserted through the most proximal hole to keep the fixator block parallel to the femur. The remaining two mounting pins were then inserted through the remaining holes. After adjusting the fixator block, a 2-mm length bone defect was created in the middle shaft of the femur using a micro drill. The mice subsequently received treatment according to their group allocations before the fascia and skin were closed by suturing using nonabsorbable thread. The surgery was radiographically confirmed using soft X-ray, and all mice were allowed to recover and move freely post-surgery. All animals were sacrificed at 4 weeks post-surgery (*n* = 6 each). The femur with external fixator was carefully dissected out for radiological evaluation and histological evaluation.

### Radiological evaluation

The process of new bone formation was monitored using soft X-ray radiographs (SOFTEX-CMB4; SOFTEX Corporation, Kanagawa, Japan) obtained using an exposure of 10 s, at 35 kV and 3.0 mA on X-Ray IX Industrial Film (Fuji Photo Film Co., Ltd., Tokyo, Japan).

### Micro-computed tomography

Femurs were fixed in 4% paraformaldehyde for 2 days at 4 °C before transferring to PBS. The tissue was imaged using a microfocus X-ray CT system (inspeXio SMX-90CT; Shimadzu, Tokyo, Japan) at a tube voltage of 90 kV, tube current of 100 μA, and voxel size of 30 × 30 × 30 μm. Images underwent 3D reconstruction using the 3D imaging software (TRI/3D BON; Ratoc System Engineering Co., Ltd., Tokyo, Japan) at a threshold determined by discriminant analysis. Bone volume (BV) and bone mineral content (BMC) of new bone at the bone defect site were calculated for all samples.

### Histological evaluation

Following micro-computed tomography, the femurs were demineralized in 20% EDTA for 4 weeks, and the remaining tissue was paraffin-embedded and cut into 3-μm-thick sagittal sections through the long axis of the femur. All sections were stained with hematoxylin and eosin (HE) and evaluated histologically. The area of new bone formed at the defect site was quantified using the freehand tracing tool in ImageJ (National Institutes of Health, Bethesda, MD) (*n* = 6).

### Statistical analysis

SPSS (version 25.0; SPSS, Chicago, IL, USA) was used for all statistical analyses. One-way ANOVA followed by Bonferroni’s post hoc comparisons test was used to analyze differences between groups, with *P* < 0.05 used to indicate statistical significance.

## Results

### Radiological evaluation

#### Soft X-ray radiography

**Fig. 1:**
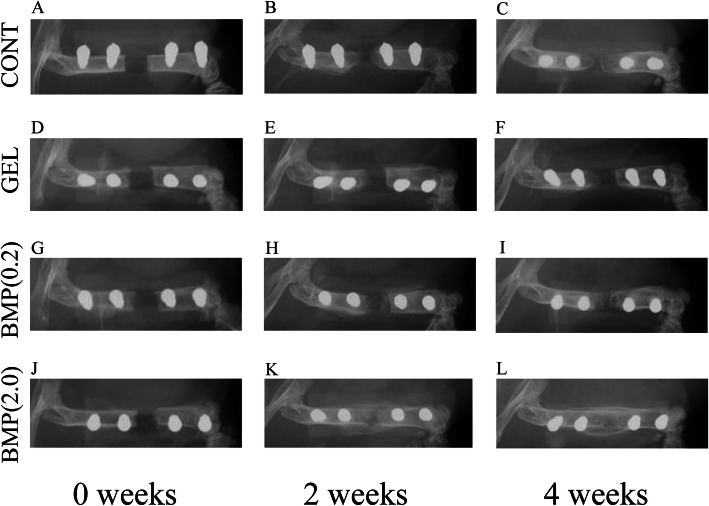
Representative time-dependent radiological changes in mouse femur bone defects. Representative radiographs of untreated and treated femurs in mice with a critical-sized bone defect. **a–c** Group CONT, **d–f** group GEL, **g–i** group BMP(0.2), and **j–l** group BMP(2.0). Radiographs were obtained immediately after surgery (**a**, **d**, **g**, **j**) and 2 (**b**, **e**, **h**, **k**) and 4 (**c**, **f**, **i**, **l**) weeks following surgery

To evaluate the time-dependent radiological changes in bone defects, we examined soft X-ray radiographs (Fig. 1a–l). In group BMP(2.0), a weak shadow of new bone was observed at the bone defect site at 2 weeks after surgery, and marked new bone formation was confirmed at 4 weeks after surgery (Fig. 1k, l). In contrast, all other groups showed nonunion at the bone defect site at 2 (Fig. 1 b, e, and h) and 4 (Fig. 1 c, f, and i) weeks after surgery.

#### Micro-computed tomography

**Fig. 2:**
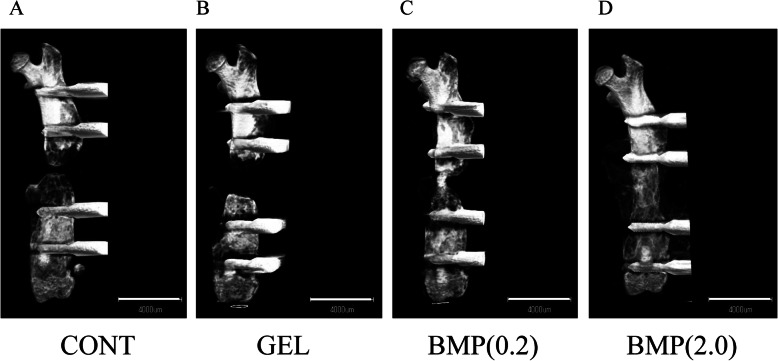
Representative micro-computed tomography images of mouse femurs at 4 weeks after surgery. 3D micro-computed tomography images of untreated and treated femurs in mice with a critical-sized bone defect at 4 weeks after surgery. **a** Group CONT, **b** group GEL, **c** group BMP(0.2), and **d** group BMP(2.0). The scale bars indicate 4 mm

**Fig. 3:**
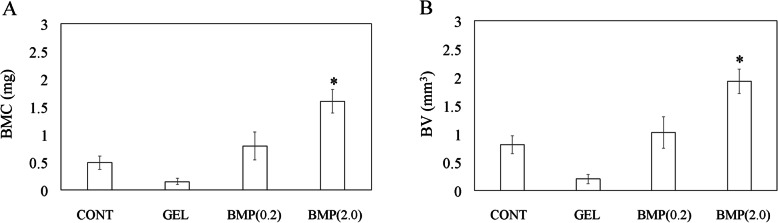
Micro-computed tomography analysis of new bone formation in mouse femurs at 4 weeks after surgery. **a** Bone mineral content and **b** bone volume at bone defect sites. Data show mean ± standard error (S.E.). *n* = 6. *P < 0.05

To assess new bone formation at the bone defect site, we examined micro-computed tomography images (Fig. 2a–d) taken 4 weeks after surgery and calculated the BV and BMC (Fig. 3a, b) in all groups. While a large amount of new bone was observed at the bone defect site in group BMP(2.0) (Fig. 2d), little new bone formation was observed in all other groups (Fig. 2a–c). In group BMP(2.0), the BV and BMC were significantly higher than those in group CONT (*P* = 0.002 and *P* = 0.003, respectively; Fig. 3a, b). In contrast, there was no difference in BV or BMC in group GEL and BMP(0.2) compared to group CONT (*P* > 0.05).

### Histological evaluation

**Fig. 4:**
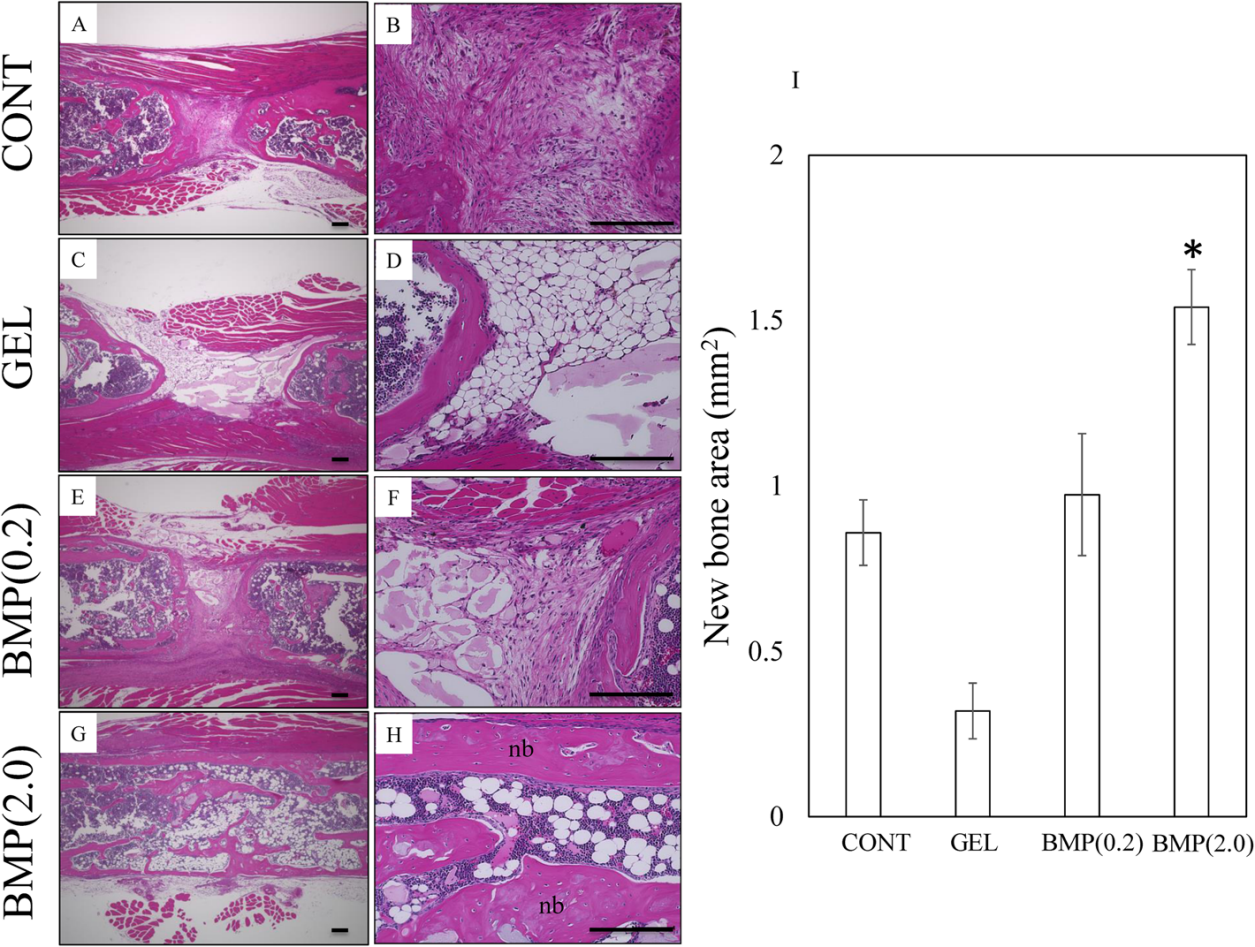
Representative hematoxylin and eosin-stained tissue sections of femurs showing new bone formation at the bone defect site at 4 weeks after surgery. Histological images showing new bone formation at the bone defect site. **a**, **b** Group CONT; **c**, **d** group GEL; **e**, **f** group BMP(0.2); and **g**, **h** group BMP(2.0). **i** Area of new bone at bone defect sites. The scale bars indicate 200 μm. Nb, newly formed bone

We also histologically evaluated the new bone formation (Fig. 4). HE staining of tissue from group BMP(2.0) showed large amounts of longitudinal trabecular bone and newly formed cortical bone at the defect site at 4 weeks after surgery, and consolidation was observed in all animals (Fig. 4h). Meanwhile, new bone formation was scarce in all other groups, with the central area between the proximal and distal bone being mainly composed of masses of fibrous and adipose tissue, and no consolidation was observed (Fig. 4a–f). The area of new bone was significantly greater in group BMP(2.0) than in group CONT (*P* = 0.007; Fig. 4i). In contrast, there was no difference in new bone area in group GEL and BMP(0.2) compared to group CONT (*P* > 0.05).

## Discussion

Several studies have examined critical-sized bone defects in the mouse femur. Some of these studies reported that 2-mm bone defects stabilized using an external fixator showing nonunions at 4 and 8 weeks after surgery [17] and were of adequate size to achieve a critical-sized bone defect at 8 weeks after surgery in mice [18]. We obtained similar results in this study in that of the control group, which received no chemical treatment, showing nonunions at the defect site at 4 weeks after surgery. In contrast, significant bone formation was observed in the 2-mm femur bone defect of mice treated with 2.0 μg of rhBMP-2 with poly(POG)n. Together, these results indicate that rhBMP-2 with poly(POG)n may be an effective material for enhancing bone repair at bone defect sites.

ACS, which is manufactured from bovine collagen type I, has been used as a BMP-2 carrier [12, 13]. We previously reported that poly(POG)n is heat resistant compared to native collagen and can be used as a carrier for growth factors [19, 20]. On circular dichroism spectral analysis, poly(POG)n showed a positive peak at 220 nm, which is indicative of a collagen triple helix, even after treatment at 80 °C [19, 20]. Poly(POG)n combined with bFGF had enhanced bone-promoting ability compared to poly(POG)n alone in a mouse fracture model [19]. Similarly, we found that rhBMP-2 with poly(POG)n accelerated bone healing in a mouse critical-sized bone defect model compared to poly(POG)n alone. BMP-2 causes mesenchymal stem cells to choose an osteogenic lineage [21], can mediate enlistment of osteoprogenitors [22] and endothelial cells [23] to defect sites, and shows high in vitro and in vivo osteoinductive activity [24]. These findings suggest that a rhBMP-2/poly(POG)n composite, which is free from animal-derived collagen, is a potential therapeutic material for enhancing bone healing in large bone defects. However, extrapolation of the results obtained from small animal models directly to humans may not be clinically relevant due to the small size of long bones, thin and fragile cortices, and lack of haversian-type remodeling in the cortex of small animals, and the dramatically higher BMP-2 doses required to induce bone repair in humans (0.1 to 1 mg BMP-2/kg body weight) [6, 25, 26]. Additional studies using large animal models (e.g., sheep, goats) are therefore important and essential for confirming our findings.

There were several main limitations in this study. First, despite showing the potential therapeutic efficacy of rhBMP-2 with poly(POG)n for treating bone defects, we did not examine the mechanisms governing the accelerated bone formation. Second, the in vitro and in vivo release kinetics of rhBMP-2 from poly(POG)n are not clear. Studies using fluorescently labeled rhBMP-2 should be performed to examine the release kinetics. Further studies are needed to gain a deeper understanding of how poly(POG)n facilitates the actions rhBMP-2 in bone healing. Finally, we did not evaluate endogenous BMP-2 expression, which plays an important role in bone healing [27, 28]. Previous study have stimulated endogenous BMP-2 expression using exogenous rhBMP-2 [29] and found that BMP-2 expression changes during the healing process. For example, treatment with rhBMP-2 stimulated BMP-2 expression on day 4 but reduced it on days 2 and 10 in a rat fracture model [29]. Further investigations are needed to evaluate endogenous BMP-2 expression at multiple time points.

## Conclusions

In conclusion, the combination of rhBMP2 and poly(POG)n gel promoted new bone formation in a critical-sized bone defect mouse model. Our findings suggest the potential of rhBMP-2 with poly(POG)n, which is free from animal-derived collagen, as a material for the treatment of large bone defects. Further studies in larger animals are needed to confirm our findings.

## Data Availability

The datasets supporting the conclusions of this article are included within the article. The raw data can be requested from the corresponding author.

## Abbreviations

rhBMP-2: Recombinant human bone morphogenetic protein-2
ACS: Absorbable collagen sponge
BV: Bone volume
BMC: Bone mineral content
HE: Hematoxylin and eosin
